# Thrombotic Complications in Pediatric Cancer

**DOI:** 10.3390/children11091096

**Published:** 2024-09-06

**Authors:** Alkistis Adramerina, Marina Economou

**Affiliations:** 1st Pediatric Department, Faculty of Health Sciences, School of Medicine, Aristotle University of Thessaloniki, 54250 Thessaloniki, Greece; marina@med.auth.gr

**Keywords:** thromboembolism, pediatric cancer, thrombophilia, anticoagulants

## Abstract

Thromboembolism (TE) complicates the course of pediatric cancer in a considerable number of cases. Cancer-related TE is attributed to an interaction of the underlying malignancy, the effects of therapy, and a possible thrombophilia predisposition. More specifically, recognized risk factors include a very young age and adolescence, non-O blood group, type and site of cancer, inherited thrombophilia, presence of central venous catheter, and type of chemotherapy. TE in children with cancer most commonly occurs in their extremities. In the absence of evidence-based guidelines for the management of thrombotic complications in pediatric oncology patients, TE management follows general recommendations for the management of pediatric TEs. Given the limitations of conventional anticoagulant therapy, direct oral anticoagulants could provide an alternative; however, their safety and efficacy in children with cancer remain to be seen. As for thromboprophylaxis, numerous studies have been conducted, albeit with conflicting results. Although the survival of pediatric oncology patients has significantly improved in recent years, morbidity due to cancer-related TE remains, underlying the need for large multicenter trials investigating both TE management with currently available agents and primary prevention.

## 1. Introduction

Cancer is characterized by a dysregulation of the coagulation system, representing a prothrombotic state. Studies focused on cancer-associated thromboembolism (TE) indicate that the local and systemic tumor cell-driven activation of the hemostatic cascade increase the thrombotic risk and contribute to the malignant process [1,2]. Firstly, malignant cells express a tissue factor that can initiate the clotting procedure after forming a complex with factor VII, while they are able to cause direct endothelial damage and subsequently platelet and tissue factor activation, leading to the upregulation of the procoagulants and the inhibition of the fibrinolytic system [1,3]. Furthermore, tumor cells interact with macrophages, resulting in the release of inflammatory cytokines, which can escalate endothelial activation. Τumors’ mechanical effects can lead to stasis and hyperviscosity, as well as vascular compression or infiltration [3,4].

Cancer-related TE is increasingly recognized in children. Children with malignancy seem to have up to a 30-fold greater risk of developing TE compared to the general pediatric population [5]. A cohort study following 498 children with cancer and 20,810 non-cancer controls concluded an absolute thrombotic risk of 1.52 and 0.06 per 1000 person-years, respectively [5]. Symptomatic or asymptomatic thrombotic complications have been reported in 16% and 40% of pediatric cancer cases, respectively [6].

Over the last few decades, the survival rate for childhood cancer has significantly improved to over 80% [7]. TE could, however, increase morbidity and even mortality [7]. A recent study in >2000 children demonstrated that TE negatively impacts the overall survival in children with high-risk leukemia [8]. TE is more frequently reported in hematological malignancies, particularly in acute lymphoblastic leukemia (ALL) and lymphoma, but also in solid tumors, such as sarcomas [7]. The pathogenesis of TE in pediatric cancer is considered multifactorial, even though it is not entirely clear yet.

## 2. Risk Factors for Thrombosis in Children with Cancer

Children with cancer are susceptible to TE through an interaction of the underlying malignancy, the effects of therapy, and a probable thrombophilia predisposition [4]. Risk factors that contribute to TE in pediatric patients with cancer can be categorized as either patient- or treatment-related [4].

- Patient-related risk factors

Children seem to face a different thrombotic risk depending on their age group. Young children, mainly under 2 years, as well as adolescents, are considered at higher risk for TE [9]. In younger ages, the smaller vasculature anatomy may be responsible for more common central venous catheter-associated thrombotic episodes, while adolescents experience more frequently aggressive cancers and their related complications, including TE [4]. Interestingly, pediatric patients with a non-O blood group present more frequently thrombotic complications, probably due to higher levels of von Willebrand factor and decreased proteolysis by ADAMTS13, compared to patients with the O blood group [9,10]. On the other hand, no gender or body mass index relation with thrombosis risk has been confirmed yet [4].

The risk for TE in pediatric cancer also depends on the diagnosis itself, as well as the site of the tumor. TE is a well-recognized, potentially severe complication of ALL in the pediatric age group. Thrombosis of the central venous system is reported in approximately 50% of TE cases, with 2% concerning pulmonary embolism and another 2% right atrium thrombosis [11]. Patients with ALL seem to present with increased von Willebrand factor antigen levels, as well as coagulation factor FVIII and FIX levels at diagnosis [12]. Moreover, reduced natural anticoagulants such as protein C have been reported [13]. These findings are attributed to the presence of peripheral blasts, which are responsible for endothelial damage and activation [14]. Thrombotic events occur, however, in combination with treatment-related risk factors, given that TE is observed mainly during induction therapy and rarely at diagnosis, unlike solid tumors [15]. In addition to ALL, children with acute myeloid leukemia, especially acute promyelocytic leukemia, experience TE more frequently [4]. Data in pediatric population remain, however, fairly limited [4]. Thrombotic events are also reported in children with lymphoma and mediastinal masses, while patients affected by solid tumors, such as sarcoma, neuroblastoma, and Wilm’s tumor, present with a higher rate of TE as well [16,17,18,19]. Any pediatric tumor with mass effect might cause extrinsic compression of neighboring vessels, altering the flow dynamics. Reduced blood flow or stasis subsequently results in TE [3,6]. Paz-Priel et al. studied 122 pediatric patients with sarcoma and reported TE in 16% of them. Approximately one third of TEs was related to tumor compression, while over half of the cases were associated with a higher disease burden and metastases [20]. Vessel infiltration of tumor cells and thrombus formation are mainly reported in patients with Wilm’s tumor [3,6].

Last but not least, inherited thrombophilia contributes to the increased risk for TE in pediatric cancer. The harmful effect of the deficiency of natural anticoagulants (antithrombin, protein C or S), the presence of the factor V Leiden gene, and even other prothrombotic defects that only pose a mild TE risk in the general population (G20210A mutation in prothrombin gene or possibly, C677T mutation in methylene tetrahydrofolate reductase) are thought to be exacerbated by the effects of the disease and its therapy in children with cancer [11]. Even though a large number of studies report on the prevalence of thrombophilia in children with ALL, their results are conflicting [4,21]. In 2003, the Prophylactic Antithrombin Replacement in Kids with Acute Lymphoblastic Leukemia Treated with Asparaginase (PARKAA) study showed no association between TE and inherited thrombophilia in ALL patients, while a trend towards an association with antiphospholipid antibodies was reported [22]. A meta-analysis, later, showed that patients with ALL and at least one thrombophilia defect face an eight-fold higher risk for TE that those without thrombophilia [23]. A more recent population-based study in Israel reported the results of 584 children with ALL tested for congenital prothrombotic disorders and concluded the presence of a significant higher occurrence of TE in patients with thrombophilia [24]. Wermes et al. evaluated inherited thrombophilia in 73 pediatric patients with ALL and 64 with other cancer forms and found that thrombophilia appeared to be an additional risk factor for TE in patients with ALL but not for children with other malignancies [25]. As very few studies evaluate the role of thrombophilia in pediatric cancer other than ALL, it is unclear whether thrombophilia screening should be routinely conducted in all pediatric patients with cancer.

b. Therapy-related risk factors

A major risk factor for the development of thrombosis in pediatric oncology patients is the presence of a central venous catheter (CVC), even though fundamental for the administration of multidrug chemotherapy and intensive support therapy. More than half of catheter-related TEs seem to be asymptomatic and their clinical relevance is currently debatable—mainly with regard to the risk for post-thrombotic syndrome, pulmonary embolism, or recurrent TE [26].

There are different types of catheter-related thrombotic complications, including intraluminal clot or tip thrombosis resulting in dysfunctional CVC, incomplete asymptomatic or complete symptomatic vein thrombosis around the lumen with patent CVC, or tip complete venous thrombosis with symptoms and dysfunctional CVC. Depending on the type, catheter-related thrombosis may lead to prolonged hospitalization and the need for urgent systematic treatment or CVC removal [27].

The vascular injury caused during insertion and the subsequent turbulent blood flow during CVC maintenance in the blood vessels increase the thrombotic risk, not only within the lumen or the tip of the catheter, but also distantly, mainly in the upper venous system. On top of that, hyperosmolar solutions, including total parenteral nutrition or chemotherapy administered through CVC, cause additional endothelial damage. Even though a pediatric study among 85 children with ALL reported that the percutaneous insertion of CVC on the left side and in the subclavian vein presented the highest TE incidence, a more recent large multicenter study in >3000 patients indicated that femoral and jugular veins were more often associated with thrombosis, so the subclavian vein should be proposed for catheter placement [28,29]. Hematological malignancy and age < 6 years have been described as significant risk factors for CVC complications (relative risk 3.0 and 2.5, respectively), while catheter-related bloodstream infection has been associated with CVC-related thrombosis as well [26,30].

On the other hand, several chemotherapy agents are shown to increase the risk for thrombosis in various ways [31]. Thus, TE may lead to therapy interruption, adversely affecting cure rates [11]. L-asparaginase (ASP), the key therapeutic agent for patients with ALL, induces a relative deficiency in the essential agent for lymphoblast survival, amino acid. However, it is considered a significant TE risk factor [11]. Thrombosis in the central nervous system is reported in up to 3% of patients during ASP treatment [23,32]. The type of ASP seems to play a role in thrombosis development. *Escherichia coli*-derived ASP, although more effective against leukemia cells, has been more frequently associated with TE than *Erwinia*-derived ASP [32]. ASP is suggested to impair the translation and secretion of hepatic proteins, including hemostatic agents such as antithrombin, protein C and S, plasminogen, and fibrinogen. Furthermore, ASP causes white cell and endothelium activation and subsequent tissue factor upregulation, promoting thrombin generation. Above all, acquired antithrombin deficiency during ASP treatment seems to predispose ALL patients to TE [33].

Steroids additionally contribute to a prothrombotic state. It is well known that they increase coagulation factors, such as prothrombin and complex factor VIII/von Willebrand factor, while impairing fibrinolytic system in a dose-dependent manner, through the elevation of plasminogen activator inhibitor 1 and a reduction in the tissue plasminogen activator [4,14]. The concomitant administration of ASP and steroids in children with ALL is associated with a higher TE risk than their separate use, with a reported adjusted odds ratio of 34.5 (95% CI: 4.39–271.42; *p* = 0.0008) [11,34].

Even if studied in a lesser extent, possible prothrombotic mechanisms have been described for anthracyclines and cisplatin, probably resulting in an increase in tissue factor activity [35,36]. Bleomycin has shown direct endothelial toxicity in animal models, while granulo-monocyte colony-stimulating factors increase thrombin activation [4,37]. Radiation therapy (RT) might be an additional risk factor for TE due to cell destruction and subsequent inflammatory prothrombotic processes, but evidence in the literature is lacking even for adults. TE incidence among adult patients who received RT was estimated at 2% [38]. The RIETE registry, which included 9284 oncology adult patients with TE, interestingly revealed an association between RT and a higher risk for cerebral bleeding following the administration of anticoagulation therapy, independently from the site of cancer and concomitant drugs [39]. Data for pediatric patients are still missing.

Intense primary chemotherapy predisposes one to more frequent and severe adverse events, including myelosuppression and infection [7]. Infection is a known independent risk factor for TE. The proinflammation state caused by pathogens and their products triggers platelet activation, leading to endothelium damage and, ultimately, thrombin generation [40]. Pathogens and pathogen-derived molecules activate also the contact-dependent coagulation pathway through direct interaction with factor XII and prekallikrein [41].

Finally, surgery and immobility can enhance the prothrombotic cancer state via endothelial damage and venous stasis. Spiegl et al., however, reported an overall TE incidence of 0.46% in 3031 major oncologic operations in pediatric cancer patients, suggesting that surgery does not put children with cancer at a significant thrombosis risk [42].

## 3. Location of Thrombosis

The majority of TEs in children with cancer occur in the venous system and are considered CVC-related [43]. In general, TEs in children with cancer most commonly occur in the upper and lower extremities. Depending on the cancer type, however, pediatric oncology patients may present TE in different locations. Cerebral thrombosis usually occurs in children with ALL. Half of the patients with ALL that experience TE present central nervous system thrombosis, resulting in acute neurological deterioration [3,44]. Cerebral sinovenous thrombosis is considered uncommon in the general pediatric population, unlike children with ALL receiving chemotherapy, who present an incidence of approximately 6% [25]. Cerebral thrombosis is described in up to 3% of patients with non-Hodgkin’s lymphoma, as well as in children with neuroblastomas [3].

Right atrial thrombosis (RAT) has been reported mainly in association with the presence of CVC’s tip in the right atrium [45]. The incidence of RAT in children with cancer and indwelling catheter is approximately 8%, although the PARKAA study revealed an even higher incidence (13.6%) in children with ALL [22,46]. Over half of the children with RAT are asymptomatic, with thrombosis incidentally detected in routine echocardiogram [3,45]. Asymptomatic, small-sized, immobile thrombi tend to have a good prognosis, irrespective of treatment; however, the removal of CVC is recommended [45]. In the absence of a CVC, RAT has been previously reported at the time of diagnosis in a patient with T-cell ALL, as well as in patients with Wilm’s tumor [47,48]. The role of RAT in pulmonary embolism development is unclear. Pulmonary embolism is more frequently associated with upper-extremity TE, and the reported incidence varies from 2% to 19.6% in children with cancer [43,49].

Portal vein thrombosis is commonly related to hepatoblastoma. Sporadic cases of portal vein thrombosis, however, have also been reported in children with other cancers, such as Burkitt lymphoma, Ewing tumor, small-cell bone tumor, or medulloblastoma in the course of chemotherapy with busulfan [50].

## 4. Management of Thrombosis in Children with Cancer

In the absence of evidence-based guidelines for the management of thrombotic complications in pediatric patients with cancer, the management of TEs in pediatric oncology patients follows the general recommendations for the management of TEs in children [51]. In 2012, the American College of Chest Physicians published guidelines for children and neonates with regard to antithrombotic therapy and the prevention of thrombosis that are still used as the standard of care. Conventional anticoagulants traditionally used in pediatric patients include heparin and vitamin K antagonists (VKAs); however, direct oral anticoagulants (DOACs) are beginning to gradually change the therapeutic landscape [52].

Unfractionated heparin (UFH) is administered in pediatric patients, although there is no therapeutic range determined for children. UFH promotes antithrombin action, resulting in anti-Xa and anti-IIa activity. Due to its short half-life, an intravenous loading dose followed by continuous infusion is required, limiting its use [15]. Adverse events include bleeding—albeit commonly resulting from accidental overdose—while osteoporosis has also been reported, so that long-term UFH administration in children is not recommended [51]. UFH is usually used for CVC flushing as prophylaxis against CVC occlusion. Even though it is common practice, the effectiveness of the method is disputed. A report including the majority of Children’s Study Group Centers in the United Kingdom showed that all centers used heparinized saline CVC flushes as thrombosis prophylaxis, despite variations in frequency, volume, and heparin concentration [53]. Ociepa et al. reported CVC thrombotic complications in 45% of the patients despite UFH flushing [54].

The anticoagulant agent of choice in the pediatric population remains the subcutaneously administered low-molecular-weight heparin (LMWH) [6]. LMWH presents no interaction with the diet or other drugs nor any association with osteoporosis [51]. In addition, close treatment monitoring is not required. The therapeutic range, extrapolated from adults receiving enoxaparin, differs from that used for UFH and is considered an anti-Xa activity between 0.5 and 1.0 U/mL, measured 4–6 h after administration in the twice-a-day regimen. Anticoagulant treatment with LMWH is generally recommended for at least 3 months, when vein recanalization occurs, and with the condition of the precipitating factor having been resolved [51].

With regard to VKAs, warfarin is most commonly used in children. The therapeutic target is considered an INR of 2.0–3.0 [51]. Even though VKAs offer the advantage of oral administration, they have other limitations, such as dietary and medication interference, as well as a narrow therapeutic window, requiring close monitoring with frequent blood tests and dose adjustments. These characteristics are exacerbated in children with a severe underlying disease or malnutrition [55]. VKAs are not even preferred in adults in the setting of malignancy [56].

DOACs provide an alternative oral anticoagulant therapy that selectively inhibits factor Xa (rivaroxaban, apixaban, edoxaban) or thrombin (dapigatran). They act independently of antithrombin, providing a wider therapeutic window, while presenting little interaction with the diet or drugs [55]. DOACs generally present a safe profile, without monitoring requirements [57]. They have been studied in completed or ongoing pediatric study trials and the results are expected to change the management of thrombosis in pediatric patients with cancer [58,59,60,61,62]. The EINSTEIN-Jr randomized trial, which evaluated rivaroxaban administration in children, included a subgroup of 56 patients diagnosed with cancer. Rivaroxaban appeared, indeed, to be safe and efficacious even in patients with anticoagulation interruptions. These positive results were not confirmed in the study by Barg et al., who reported on real-world data from 16 children with cancer-associated thrombosis receiving rivaroxaban. Even though satisfactory thrombus resolution rates were described, higher rates of thrombotic and bleeding complications than usually observed were reported, especially in children with a high disease burden [63]. DOACs’ safety in the management of thrombosis in pediatric cancer needs to be proven in larger studies.

Outside the study setting, one has to note that, despite being an unfortunate cause, the COVID-19 pandemic has added much information in terms of the overall experience of anticoagulant use in pediatric patients, and, along with the rest of pediatric thrombosis patients, this gained experience will benefit children with cancer, enabling them to receive the anticlotting agent which fits them best.

## 5. Thromboprophylaxis in Pediatric Cancer

Thromboprophylaxis in children with cancer has been a subject of discussion for years. Numerous studies—mainly including children with ALL—have been conducted, with conflicting results. Starting in 2003, the PROTEKT trial aimed to evaluate the safety and efficacy of LWMH versus standard-of-care treatment in the prevention of CVC-related thrombosis in 186 children. Half of the patients enrolled were diagnosed with cancer. Even though the study was prematurely closed due to slow recruitment, no difference in the TE incidence was reported in the two arms [64]. At that time, the PAARKA study evaluated prophylactic antithrombin replacement in children with ALL and CVC during the induction phase, only showing a trend towards efficacy [32]. Later on, Mitchell et al. developed a predictive model with four variables (type of steroids, concomitant use of ASP and prednisone, thrombophilia, and CVC) in order to identify children with ALL at high risk for TE. The model was validated in 339 children, indicating 96.2% specificity, but only 63.2% sensitivity [65]. In 2017, Tropic ALL was announced as the Dutch multicenter, randomized controlled trial assessing thromboprophylaxis with LMWH versus standard-of-care treatment in children treated for ALL. However, the results are still pending [66]. Moreover, the THROMBOTECT study prospectively compared the safety and efficacy of prophylaxis with low-dose UFH, LMWH, or an antithrombin replacement in 949 children with ALL. Although one third of the patients in the LMWH arm refused thromboprophylaxis, enoxaparin was proven more effective as a preventive strategy [67].

Finally, a meta-analysis by Pelland-Marcotte, studying multiple thromboprophylaxis alternatives in pediatric patients with cancer, resulted in the superiority of LMWH over VKAs, antithrombin replacement, or standard-of-care treatment. The data, however, concerned mainly children with ALL (97.5%) [68].

Furthermore, PREVARIX-ALL, a randomized controlled study assessing primary thromboprophylaxis with apixaban versus no thromboprophylaxis in children with ALL or lymphoma, showed no treatment benefit for patients receiving apixaban. In fact, the apixaban arm presented a higher incidence of clinically relevant non-major bleedings [69].

Recently, Athale et al. reported on clinical and laboratory predictors of TE in children with ALL. An age over 10 years, the white blood cell count at diagnosis, T-ALL, high-risk ALL, and a non-0 blood group were identified as risk factors for TE, but, interestingly, not single-nucleotide polymorphisms for prothrombin G20210A and factor V G1691A. Age was defined as an independent TE predictor, so thromboprophylaxis was suggested for consideration in all children over 10 years of age [70].

Despite all which has been learnt over the last few decades, the optimum approach for TE prevention in pediatric cancer still needs to be determined with further studies. The identification of all possible—and, even more so, the most probable—factors associated with an increased risk for cancer-associated thrombosis could allow for a more individualized approach.

## 6. Conclusions

Thanks to recent advances in diagnosis and treatment, more and more children are surviving cancer types that were previously considered fatal. As a consequence, they experience disease- and/or treatment-related complications. Symptomatic and asymptomatic TE is one of these complications, resulting in considerable morbidity among pediatric cancer patients, most commonly in children with hematological malignancies. TE management can be challenging in these patients, especially if thrombocytopenia is present.

Thromboprophylaxis in children with cancer could be of great benefit; however, the available data are unclear regarding the patient group that would benefit the most. LMWH has proven safe and effective as prophylaxis, while DOAC use could overcome the limitations of heparin administration, if proven to be comparable in terms of efficacy and safety. Large, multicenter trials investigating TE management with all currently available agents, as well as primary TE prevention, are of great importance, as these young patients are expected to live for many decades following a thrombotic event.
